# Experts' discussion: implications of the World Health Organization's World report on hearing for the cochlear implant field

**DOI:** 10.1016/j.bjorl.2024.101556

**Published:** 2025-01-03

**Authors:** Wolf-Dieter Baumgartner, Javier Gavilán, Abdelhamid Benghalem, Suela Sallavaci, Gunesh Rajan, Ranjith Rajeswaran, Mario Zernotti, Shelly Chadha

**Affiliations:** a Vienna ENT University Hospital, Vienna, Austria; b La Paz University Hospital, Department of Otorhinolaryngology, Madrid, Spain; c Clinique Rachidi, ENT Department, Casablanca, Morocco; d University Hospital Centre “Mother Theresa”, Department of Otorhinolaryngology, Tirana, Albania; e Lucerne Cantonal Hospital, Department of Otolaryngology, Head and Neck Surgery, Lucerne, Switzerland; f Madras ENT Research Foundation (MERF), Audiology Department, Chennai, Tamil Nadu, India; g Sanatorio Allende de Córdoba, Department of Otorhinolaryngology, Córdoba, Argentina; h World Health Organization, Department of Noncommunicable Diseases, Rehabilitation and Disability, Disability and Rehabilitation Unit, Geneva, Switzerland

**Keywords:** Cochlear implants, Hearing loss, World Health Organization, Accessibility, Social stigma

## Abstract

**Objective:**

This review aims to analyse the implications of the World Health Organization's 2021 world report on hearing, with a particular focus on the cochlear implant field. The objective is to understand the challenges and opportunities highlighted in the report and propose viable solutions for effective implementation within the cochlear implant community.

**Methods:**

Following the release of the World Health Organization's world report on hearing, cochlear implant professionals explored and discussed the implications of the report with examples from various countries to understand the disparities in access, reimbursement policies, and social stigma associated with hearing loss.

**Results:**

The world hearing report identifies hearing loss as a major global health issue, with an estimate of 1.5 billion people currently affected and a projected increase to 2.5 billion by 2050. Key challenges in the cochlear implantation field include disparities in access to services, particularly in low- and middle-income countries, lack of reimbursement policies in many regions, and the social stigma surrounding hearing loss.

**Conclusion:**

A multi-faced approach is needed to address the rising prevalence of hearing loss and policy makers must prioritize ear and hearing care as a public health concern. Organizations like the HEARRING group can promote education, awareness, and training, and act as advocates for changes in healthcare systems to expand access to hearing care services.

## Highlights

• Hearing loss is on the rise.
• Low- and middle-income countries face more challenges.
• Key challenges are access disparities and lack of reimbursement policies.
• Hearing care should be integrated into the national primary healthcare.
• Organizations like HEARRING can promote education and awareness.

## Introduction

This review analyses the implications of the World Health Organization's (WHO) world report on hearing focusing on the Cochlear Implant (CI) field. This matter is of particular importance as hearing loss has been identified as a major global health issue with vast implications for individuals and society. The WHO report, released in 2021, provides a comprehensive review of the global state of hearing and proposes strategies for progress.^1^ In response to this report, CI professionals are examining the implications of the WHO report for the CI field, addressing associated challenges, and proposing viable solutions for effective implementation. Key challenges outlined include disparities in access to hearing care services, particularly in low- and middle-income countries, an absence of reimbursement policies in many regions, and the social stigma surrounding hearing loss. An encompassing approach, therefore, is necessary to address the escalating prevalence of hearing loss. The review also depicts how organizations such as HEARRING can play a crucial role in establishing education, awareness, and training, which are imperative for a rounded approach to hearing loss and ear disorders.

## Hearing loss on the rise

Hearing care is essential for promoting effective communication, emotional well-being, safety, educational and vocational success, and an overall improved quality of life. Moreover, untreated hearing loss has a substantial economic impact on both individuals and society as a whole because it can lead to reduced productivity at work and increased healthcare costs.^2^ Hearing is also an integral part of cognitive function. Studies have linked untreated hearing loss to an increased risk of cognitive decline, including conditions like dementia and Alzheimer's disease.^3^, ^4^, ^5^ Therefore, treating hearing loss in the elderly population has far-reaching implications, positively influencing their cognitive abilities, social interactions, and emotional state and mitigating conditions such as depression and dysphoria. Considering the global trend of an ageing population, it is imperative to prioritize hearing care and proactively address hearing loss.^6^

Hearing loss is increasingly prevalent not only among the elderly but across all age groups. Currently, an estimated 1.5 billion people worldwide are affected by hearing loss, with nearly one third of this cohort requiring hearing rehabilitation services.^1^ By the year 2050, the global population is expected to reach 10 billion individuals. According to WHO’s estimations, a substantial proportion, approximately 2.5 billion people, will have some degree of hearing loss, ranging from mild to profound or complete hearing loss, and of those over 700 million will benefit hearing rehabilitation services.^1^

While demographic characteristics undoubtedly contribute considerably to the growing prevalence of hearing loss, it is necessary to recognize the increase in modifiable risk factors. For instance, exposure to excessively loud auditory stimuli is a known hearing loss risk factor, nevertheless over a billion young people today are at risk of hearing loss simply because of the way they are experiencing music.^7^ Moreover, easily treatable and in part preventable ear infections continue to affect up to 200 million people worldwide, often resulting in hearing loss and other life-threatening complications.

### Challenges

The vast majority, nearly 80%, of people with hearing loss, live in low- and middle-income countries, where ear and hearing care services are limited or practically non-existent. According to the WHO hearing report, only approximately 17% of people who could potentially benefit from hearing technology actually obtaining it. This disparity varies dramatically, ranging from 25% in high-income countries to over 10% in low- and middle-income countries.^1^ This chasm in hearing care access comes at a substantial cost, both to the individuals affected with hearing loss and to society as a whole. It has been estimated that the world loses nearly a trillion dollars annually due to diminished productivity attributed to the social isolation stemming from unaddressed hearing loss.^8^

While there are notable success stories in countries like Switzerland, where free and universal neonatal hearing screening is effectively implemented, both children and adults are able to receive bilateral CIs when needed, and where there is a dedicated disability insurance system for people with hearing impairment,^9^ low- and middle-income countries still face many challenges. In Latin America for instance, a mere 20%–25% of those in need of a hearing aid or CI have access to these essential technologies. In Argentina, approximately 700 CIs are implanted each year, but only in 20% of these cases is the cost covered by the government, with the remaining relying on private insurance or donations. The situation is even direr in countries like Bolivia and Paraguay, where the government support for CIs is non-existent. The significant challenge in treating people with hearing loss lies in the high associated costs. A standard hearing aid alone can amount US$1000, while a cochlear implant generally exceeds US$10000.^10^ Moreover, there are additional expenses related to the implementation of audiological diagnosis programs, rehabilitation, specialist training, and professional remuneration. Considering the projected increase in the number of individuals with hearing loss worldwide in the following years, it is evident that the costs will surpass the available resources.^10^ Consequently, it is imperative to reconceptualize a new model for diagnosing and treating people with hearing loss. Regrettably, several Latin American countries, such as Venezuela, Paraguay, Bolivia, Nicaragua, El Salvador, and most of the countries in the Caribbean region, lack systematic protocols to early detection, coverage, and reimbursement of prosthetic devices.^11^

A similar situation is observed in the Balkan region. In Albania, the first screening programs for children and newborns took place twenty years ago and were funded either privately or through university programs. It was not until 2020 that hearing care advocates were able to secure government funding for all newborn screening and an additional support for 20 CIs annually. However, the prevalence of profound hearing loss in Albania, based on current screening data, stands at 0.2%.^12^, ^13^ This means that approximately 60 children with profound hearing loss are born each year in Albania, but only a third of them receive the necessary funding for a CI. The only option for the other two thirds is to be treated privately where all expenses must be covered by the patients. Moreover, there is no reimbursement for CIs for adolescents and adults. A similar situation existed in Morocco, where cochlear implantations commenced twenty years ago, initially relying solely on charitable donations for support. Following a challenging period of 5- to 7-years without any government support, hearing care advocates were able to leverage their accomplishments to establish a reimbursement program that now covers a remarkable 70% of the cost of CI, either through government funding or private insurance.^14^, ^15^, ^16^ Another interesting case is that of India, which has just over 30 states and where financial healthcare policies are a state rather than a federal subject. Until 2008, pediatric CIs were entirely self-funded, however, now CIs for children below the age of 12 are reimbursed by the federal government. Unfortunately, individuals above the age of 12 receive no such reimbursement, either from the government or from their insurance providers. The only possible reimbursement for them is through the Corporate Social Responsibility (CSR) initiatives, charitable donations, or by submitting a special request to the Chief Minister or Prime Minister of the country, who may grant CIs on a case-by-case basis.^17^, ^18^

There is often a social stigma associated with hearing loss, and the extent of this stigma can vary depending on multiple factors, including the cultural, socioeconomic, and educational context.^19^ Stigma can be fuelled by misconceptions about the causes of hearing loss and a lack of awareness about available treatments. Cultural beliefs and traditions may also influence how hearing loss is perceived. For example, some cultures tend to hide disabilities, and this is even more pronounced in small villages and remote areas where people are more attached to tradition. Stigma can exist in low-, middle-, and high-income countries, but the specific factors contributing to it and how it manifests may differ. Limited access to healthcare and especially ear and hearing care, can result in a higher prevalence of untreated hearing loss. In Morocco for instance, the social acceptance of hearing aids and CIs has been a challenging process and raising awareness has been very difficult. It required a multifaced approach, starting with efforts to involve families and the wider community, and extending to attempts to gain support from both governmental and non-governmental organizations.^15^

The prevalence of hearing loss and attitudes towards it can vary widely even within a country. Urban areas may have better access to healthcare and education, leading to reduced stigma, while rural areas may suffer in terms of awareness and services. A good example of this is India, where many districts have hearing healthcare, but it does not reach everyone, and especially people in remote areas. In high-income countries, hearing loss stigma still exists but may be somewhat reduced by increased awareness, better access to healthcare, and anti-discrimination laws. Nevertheless, even in countries where hearing aids or CIs are free, people delay seeking treatment for hearing loss by as much as 7- to 10-years.^1^ Therefore, in every society still exist stereotypes and misconceptions about hearing loss, exposing people with hearing loss to discrimination and social isolation and creating challenges to obtaining the same levels of education and employment enjoyed by people without functional hearing loss.

### Solutions

Public health action can help people hear, regardless of their age. Actions that focus on effective public health strategies and clinical interventions that can prevent the onset and progression of hearing loss. According to the WHO hearing report, nearly 60% of hearing loss in children is due to preventable causes.^1^, ^20^

Early identification of hearing loss is crucial. Screening services for newborns, children, and adults need to be integrated into public health. Various innovative screening solutions exist and can be tailored to cover the needs of each region, with tele-audiometry and tele-medicine models reaching even the remotest parts of the world.

Efforts to eliminate the stigma of hearing loss may involve education, awareness campaigns, and policies that promote accessibility and inclusion. Awareness should be raised in different levels and by various approaches. First and foremost, efforts must be made at the family and community level in order to eliminate any misconceptions and stereotypes and to provide sufficient information to people about their options of treating hearing loss. Examples from India and Albania show that going directly to remote communities is an effective way of building an awareness and referral system so that people will know where to go and what to do when they need ear and hearing care.^21^, ^22^, ^23^ Such direct approaches provide solutions that are individually suited to specific regions and cultures. Consequently, they present an effective way of targeting deep-rooted cultural stereotypes associated with hearing loss. On a next level, awareness can be raised in the society through the educational system by including, for instance, a story about hearing loss for children in the kindergarten or primary school, or through the use of social media by creating and promoting advocacy groups for CI users (and their families), since they are the best ones to directly show their progress and results and inspire other people dealing with hearing loss to take the first step and ask for help. In Europe, the European Association of Cochlear Implant Users (EURO-CIU a.s.b.l.),^24^ a non-governmental and non-profit association founded almost 30-years ago, consists of 33 national member associations from 25 European countries and aims to raise awareness and support CI users. On an international level, Hearpeers Mentors is a growing global online community that allows candidates to get in touch with experienced CI users from their region to access information and share personal experiences.^25^ A number of smaller online groups can be found on social media where CI users and candidates can freely join and discuss with each other. Small unofficial groups like these could help to fight the social stigma and break stereotypes, while larger organized association and communities can raise awareness, support CI users, and put pressure on policy makers to promote CIs.

**Fig. 1:**
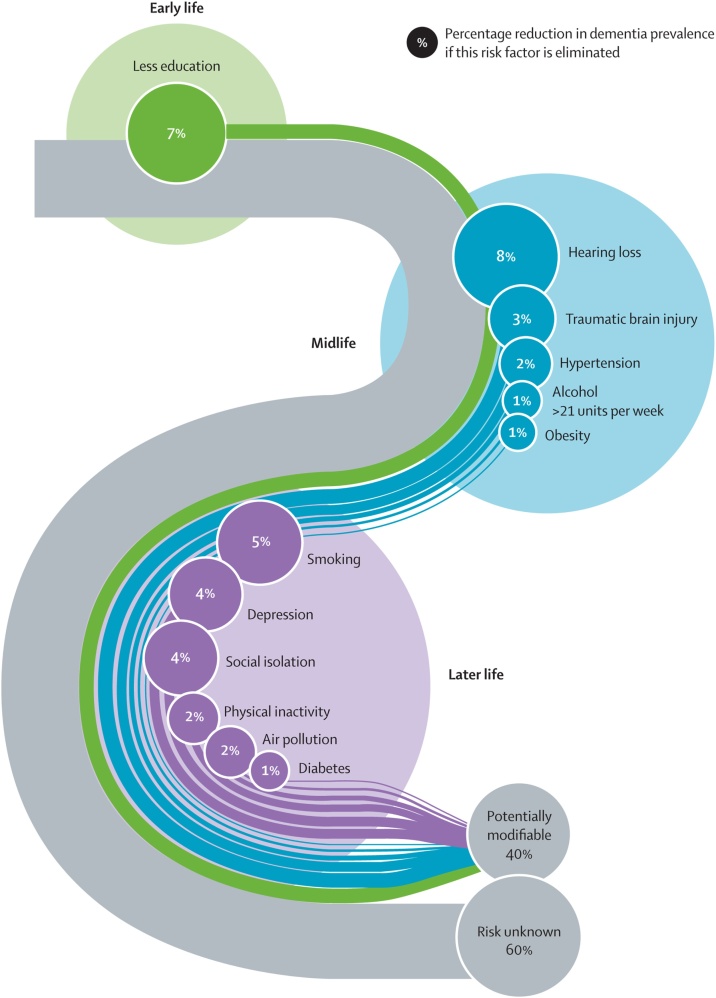
Proportion of dementia cases attributable to modifiable risk factors according to Livingston G et al., 2023 (Copyright 2023, Elsevier; Licence for reprinting acquired from Elsevier).^26^

At the same time, professionals must become advocates for public health measures for ear and hearing care, and especially for its integration into the national primary healthcare system so that is freely accessible to every child and adult, even in the remotest parts of the world. Pressure must be put on policy makers and governments to act. The WHO world hearing report predictions can be used to create realistic models, customized for each country, to provide information on how much money will be lost in the next few years if nothing is done about ear and hearing care. Moreover, hearing loss is the only contributor to dementia that can be treated. According to the Lancet commission report on dementia, adult hearing loss is the most common modifiable risk factor for dementia (Fig. 1).^26^ Treating hearing loss can also impact other dementia-associated risk factors, such as social isolation.

Overall, it is fundamental to put ear and hearing care on the political agenda by using all available data and constantly and persistently reaching out to policy makers. Additionally, strategic timing is crucial in creating momentum and moving things forward, for instance the period leading up to elections presents an ideal opportunity to advocate for these critical needs. Raising awareness and addressing the stigma of hearing loss is a global challenge that requires collaboration between governments, healthcare organizations, advocacy groups, and the public to create more inclusive and accepting societies for individuals with hearing loss.

## Conclusion

The WHO hearing report states that “the cost of doing nothing is one we cannot afford”.^1^ In response, it proposes a framework for people-centered ear and hearing care, integrated in the primary healthcare system. This framework is based in a series of interventions that countries should consider in the light of their specific needs and then deliver the necessary changes to strengthen their health system. The HEARRING group can be a valuable tool in advancing these ideas through its focus on promoting education, awareness, and training; the three pillars for a comprehensive approach to the problem of hearing loss and ear disorders. It can provide the necessary education to non-ENT physicians and create efficient referral pathways, as well as act as facilitator for the creation of high-quality hearing implant programs for the training of ENT physicians and especially audiologists and surgeons. Moreover, the HEARRING group can contribute to raising awareness at various different levels, including engaging with funding agencies and ministries of health, to advocate for the universal implementation of neonatal screening, as well as screening programs for adults and the elderly, and ensuring the reimbursement of CIs and hearing aids. Finally, HEARRING can push for changes to expand the indications,^27^ thereby allowing more people affected by hearing loss to benefit from life-changing intervention.

## CRediT authorship contribution statement

Conceptualization, JG, WDB; Methodology, n/a; Software, n/a; Validation, n/a; Formal analysis, n/a; Investigation, n/a; Resources, n/a; Data curation, n/a; Writing–Original draft, AB, GR, JG, MZ, RR, SC, SS, WDB; Writing-Review and editing, AB, GR, JG, MZ, RR, SC, SS, WDB; Visualization, n/a; Supervision, n/a; Project administration, n/a; Funding acquisition, none. All authors have read and agreed to the published version of the manuscript.

## Informed consent statement

Not applicable.

## Ethical approval

Not applicable.

## Funding

The author(s) received no financial support for the research, authorship, and/or publication of this article.

## Meeting information

Discussion of the WHO report on hearing, HEARRING Group, 17 May 2021.

## Data availability statement

There are no data presented in this paper as this is based on a panel review.

## Declaration of competing interest

The authors declare no conflicts of interest.

## References

[1] World Health Organization (2021). https://www.who.int/publications/i/item/9789240020481.

[2] Huddle M.G., Goman A.M., Kernizan F.C. (2017). The economic impact of adult hearing loss: a systematic review. JAMA Otolaryngol Head Neck Surg..

[3] Lin F.R., Yaffe K., Xia J. (2013). Hearing loss and cognitive decline in older adults. JAMA Intern Med..

[4] Calvino M., Sánchez-Cuadrado I., Gavilán J., Gutiérrez-Revilla M.A., Polo R., Lassaletta L. (2022). Effect of cochlear implantation on cognitive decline and quality of life in younger and older adults with severe-to-profound hearing loss. Eur Arch Otorhinolaryngol..

[5] Babajanian E.E., Gurgel R.K. (2022). Cognitive and behavioral effects of hearing loss. Curr Opin Otolaryngol Head Neck Surg..

[6] Ellis S., Ali S.S., Ahmed W. (2021). A review of the impact of hearing interventions on social isolation and loneliness in older people with hearing loss. Eur Arch Otorhinolaryngol..

[7] Natarajan N., Batts S., Stankovic K.M. (2023). Noise-induced hearing loss. J Clin Med..

[8] Chadha S., Kamenov K., Cieza A. (2021). The world report on hearing, 2021. Bull World Heal Organ..

[9] Brand Y., Senn P., Dillier N., Kompis M., Allum J. (2014). Cochlear implantation in children and adults in Switzerland. Swiss Méd Wkly..

[10] Délano P. (2019). Hearing aids: a solution for all? [Dispositivos de ayuda auditiva: Una solucin para todos?]. Rev Otorrinolaringol Ciruga Cabeza Cuello..

[11] Berruecos P. (2021). Cochlear implants in Latin America [Los implantes cocleares en América latina]. Auditio..

[12] Kalcioglu M.T., Sallavaci S., Hrncic N. (2021). Prevalence of and factors affecting otitis media with effusion in children in the region from Balkans to Caspian basin; A multicentric cross-sectional study. Int J Pediatr Otorhinolaryngol..

[13] Suela S., Ervin T., Ylli S., Gentian S. (2014). Prevalence of hearing loss among first grade school children in Tirana,Albania – A repeated cross-sectional survey. Otolaryngol: Open Access..

[14] Agence Nationale de l’Assurance Maladie. National reference rates for reimbursement or coverage of medical equipment and devices [Tarifs nationaux de référence pour le remboursement ou la prise en charge des appareillages et dispositifs médicaux]. Accessed April 24, 2024. https://www.cnss.ma/sites/default/files/Remboursement%20et%20prise%20en%20charge%20et%20appareillage%20des%20dispositifs%20m%C3%A9dicaux.pdf.

[15] Le Ministère de la Solidarité, de Développement Social, de l’Egalité et de la Famille. National Programme for the Diagnosis and Management of Children and Young People with Hearing Disabilities and Deafness [Programme National de diagnostic et de prise en charge des enfants et des jeunes en situation de handicap auditif et de surdité]. Accessed April 24, 2024. https://social.gov.ma/personnes-en-situation-de-handicap-personnes-handicapees/programme-national-de-diagnostic-et-de-prise-en-charge-des-enfants-et-des-jeunes-en-situation-de-handicap-auditif-et-de-surdite-programme-nasmaa/.

[16] Royaume du Maroc, Ministère de la Santé, Direction de la Population. Guidelines for Screening and Management of Deafness in Children [Référentiel de dépistage et de prise en charge de la surdité chez l’enfant]. Accessed April 24, 2024. https://www.sante.gov.ma/Publications/Guides-Manuels/Documents/2022/Re%C4%9Bfe%C4%9Brnetiel%20de%C4%9Bpistage%20n%C3%A9onatal%20de%20la%20surdit%C3%A9%20chez%20l%27enfant%20publi%C3%A9.pdf.

[17] Central Board of Secondary Education. Reimbursement of Cost of Cochlear Implant. https://www.cbse.gov.in/cbsenew/personnel_cir/cost%20of%20cochlear%20implant%20-%2014.01.2020.pdf.

[18] Office of the Principal District & Sessions Judge: Delhi. Handbook for Medical Reimbursement (3rd Edition) on Delhi Government Employees Health Scheme (D.G.E.H.S). Accessed April 24, 2024. https://delhicourts.nic.in/medical%20reimbursement_0001.pdf.

[19] Wallhagen M.I. (2010). The stigma of hearing loss. Gerontol..

[20] Chadha S., Cieza A. (2018). World Health Organization and its initiative for ear and hearing care. Otolaryngol Clin North Am..

[21] Chadha S. (2013). Increasing community awareness of ear and hearing health. Community Ear Hear Heal..

[22] Ricard P. (2013). Raising awareness to empower communities to take action. Community Ear Hear Heal..

[23] Aitken M., Backliwal A., Chang M., Udeshi A. (2013). http://www.imshealth.com.

[24] European Association of Cochlear Implant Users (EURO-CIU a.s.b.l.). Accessed April 24, 2024. https://eurociu.eu/.

[25] Hearpeers Mentors. Accessed April 24, 2024. www.hearpeers.com.

[26] Livingston G., Huntley J., Sommerlad A. (2020). Dementia prevention, intervention, and care: 2020 report of the Lancet Commission. Lancet.

[27] Van de Heyning P., Gavilán J., Godey B. (2022). Worldwide variation in cochlear implant candidacy. J Int Adv Otol..

